# Analysis and optimization of inpatient cost structure for fracture patients under the implementation of the DRG policy

**DOI:** 10.3389/fpubh.2025.1648606

**Published:** 2025-12-01

**Authors:** Changgeng Su, Jiuhui Su, Xin Li, Xinyan Jin, Zhiyue Wang, Jiwei Liu, Hongjuan Wen

**Affiliations:** 1 College of Health Management, Changchun University of Chinese Medicine, Changchun, Jilin Province, China; 2 Haicheng Orthopedic Hospital, Haicheng, Liaoning, China; 3 Monitoring and Statistical Research Center of the National Administration of Traditional Chinese Medicine, Beijing, China

**Keywords:** drug, fracture, inpatient cost, analysis and optimization, interrupted time series, gray relational analysis, health policyoptimization

## Abstract

**Background:**

Fractures are among the most common traumatic injuries in China, with rising incidence driven by population aging, traffic accidents, and sports injuries. They impose a heavy economic burden due to high treatment costs and prolonged rehabilitation. To improve cost efficiency, China launched a nationwide Diagnosis-Related Groups (DRG) payment reform in 2019. While DRG has shown positive effects in controlling costs for chronic diseases, its impact on trauma-related conditions like fractures remains unclear due to clinical complexity and treatment variability. Existing research mostly focuses on epidemiology, lacking economic evaluations under the DRG system. This study aims to assess how DRG reform influences the inpatient cost structure of fracture patients and explore differential effects across comorbidity and fracture types, providing evidence for more refined payment strategies in trauma care.

**Methods:**

Using data from 12,101 hospitalized fracture patients (ICD-10 codes S22–S92) admitted to a tertiary hospital in Anshan, Liaoning Province between 2018 and 2024, we conducted a structural change analysis to assess shifts in the composition of inpatient costs before and after the introduction of the DRG payment system. An interrupted time series (ITS) model was applied to estimate both the immediate impact and the longitudinal trend changes associated with the DRG reform initiated on July 1, 2019. In addition, gray relational analysis was employed to further examine the relative contribution of different cost categories to overall expenditure.

**Results:**

A total of 12,101 fracture inpatients were included, covering the entire period before and after the DRG reform. Significant changes in the cost structure were observed post-implementation. The median drug cost decreased from 3,416.06 CNY to 2,796.74 CNY (a reduction of 18.1%). Although the proportion of consumables costs slightly increased (median rose from 7,358.12 CNY to 7,465.64 CNY), the growth rate significantly slowed (*p* < 0.05). Meanwhile, therapeutic costs (median increased from 1,015.38 CNY to 1,200.91 CNY) and the proportion of surgical fees rose, indicating a shift of medical resources toward technical services under DRG. Rehabilitation costs declined in certain fracture types (e.g., femoral fractures, S72), but increased in others (e.g., lower leg fractures, S82), reflecting DRG’s differential effects on treatment stages. Structural variation analysis showed the greatest fluctuation in consumables costs in spinal fracture cases (S32 group, DsV = 2170.42%), while drug costs significantly declined in the S72 group (DsV = −39.78%). Patients with comorbidities experienced more pronounced structural adjustments—for example, the structural variation in the hypertensive group was 15.3% higher than that in the non-comorbidity group (*p* < 0.01), suggesting stronger cost-control effects of DRG in complex cases. ITS analysis revealed that the DRG reform had a significant impact on costs across various fracture types (*p* < 0.05). For total costs, S32 fractures exhibited a reversal from a pre-policy increasing trend (β₁ = 1247.93) to a rapid decreasing trend (β₃ = −2467.0). After the DRG implementation, diagnostic costs showed an increasing trend in most fracture types, while decreasing significantly in S32 fractures (β₃ = −227.16); in contrast, S72 fractures demonstrated a notable increase (β₃ = 52.86). Treatment and medication costs generally displayed decreasing trends, with the most pronounced decline observed in medication costs for S32 fractures (β₃ = −355.1). Consumables costs exhibited a divergent pattern, characterized by an anomalous increasing trend in S42 fractures (β₃ = 1578.62). Rehabilitation costs showed a universal decreasing trend, with the most significant control effect seen in S32 fractures (β₃ = −483.58). Gray relational analysis indicated that, before and after DRG implementation, the cost struetures of different fracture types exhibited distinct patterns of change. In S22 and S42 fractures, the correlation coefficients of diagnostic and drug-related costs increased notably, with all categories in S42 rising to 0.89–0.90, reflecting a highly concentrated cost structure. S32 and S82 fractures showed overall stability or slight increases across cost categories. In contrast, S52 and S62 fractures demonstrated a general decline, particularly in therapeutic, consumable, and rehabilitation costs. S72 fractures remained relatively stable, with minimal fluctuations in correlation coefficients. S92 fractures displayed increases across all cost categories with balanced magnitudes, indicating a comprehensive enhancement. Overall, different fracture types exhibited distinct patterns of cost structure adjustment following DRG implementation.

**Conclusion:**

The DRG-based payment reform effectively controlled pharmaceutical expenditures while increasing diagnostic costs, leading to fracture-type-specific shifts in treatment structure and highlighting the need for differentiated management strategies. This study provides empirical evidence to support the optimization of DRG payment standards and the advancement of healthcare payment reform.

## 1 Introduction

In China, trauma is the fifth leading cause of death across the entire population (1), and fractures are among the most common traumatic conditions. Their incidence is closely linked to the ongoing process of population aging. As the proportion of older population continues to rise, the incidence of age-related fractures—such as osteoporotic and hip fractures—has increased significantly. Meanwhile, factors such as traffic accidents and sports injuries have also contributed to the persistently high fracture rates among younger and middle-aged populations. According to the Global Burden of Disease Study (2), there were 178 million new fracture cases worldwide in 2019, representing a 33.4% increase compared to 1990. The number of individuals suffering from acute or chronic symptoms related to fractures reached 455 million, with a 70.1% increase over the same period (2). It is projected that by 2050, the annual number of hip fractures among older population in China will reach 1.3 million (3) Fractures often lead to absenteeism, reduced productivity, disability, impaired quality of life, substantial health losses, and high medical costs (4–7). To address these challenges, health insurance payment reform in China is imperative. Against this backdrop, Diagnosis-Related Groups (DRG) payment reform has emerged as a critical breakthrough in promoting refined healthcare management and improving medical efficiency (8, 9).

The DRG payment system originated in the United States in the 1960s (10). Its core principle is to classify diseases into distinct groups based on diagnosis, treatment methods, complications, and other factors, with each group assigned a fixed reimbursement rate—thus achieving the goal of “equal treatment and equal payment for the same disease.” Over the past two decades, several countries have adopted prospective DRG-based payment systems (11, 12). In China, a national DRG payment pilot was officially launched in 2019, initially covering 30 cities and gradually expanding nationwide (13, 14).

Current research on the impact of DRG payments on healthcare systems predominantly focuses on chronic diseases (15–19), generally confirming that its implementation can effectively reduce hospitalization costs, shorten average length of stay, and curb unnecessary medical interventions. However, in disease areas such as fractures, which are characterized by high rates of emergency surgery, prolonged recovery periods, and significant individual variability, the standardized DRG payment model faces particular challenges (20–22). Although progress has been made in epidemiological studies of traumatic fractures in China (23–26), systematic research on economic burden analysis—particularly within the context of DRG payment—remains notably underdeveloped. Specifically, the current DRG payment mechanism primarily covers the inpatient phase and struggles to adequately account for the extended costs associated with fracture patients, such as long-term rehabilitation needs, multiple follow-up visits, and secondary surgeries (27–31). This institutional design may lead to insufficient incentives for healthcare institutions when treating patients with complex fractures, thereby undermining their motivation to provide systematic and continuous medical services. Therefore, there is an urgent need to conduct research on the effectiveness of payment policies tailored to the unique spectrum of fracture-related conditions, in order to provide evidence for developing a refined medical insurance payment model that aligns with their clinical characteristics.

This study integrates interrupted time series analysis (ITS), structural variation metrics, and gray relational analysis to investigate the heterogeneous impact of the DRG payment system on fracture patients across different diagnostic groups. The aim is to provide evidence-based recommendations for developing differentiated medical cost-control strategies and to support the ongoing reform and optimization of China’s healthcare payment system.

**Figure 1:**
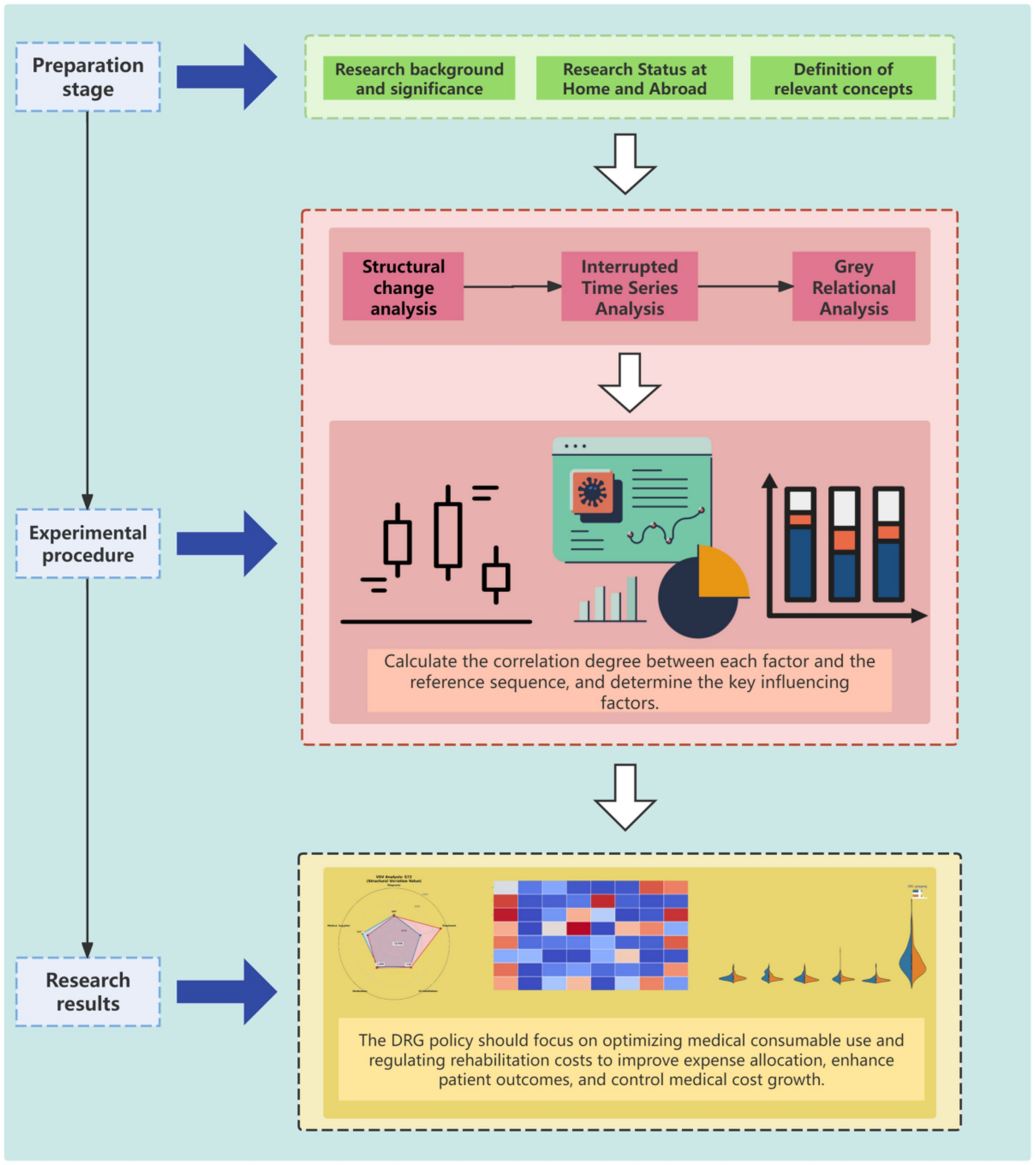
Flowchart of the study design and analysis process.

A schematic of the study design is presented in Figure 1.

## 2 Methods

### 2.1 Data

The data for this study were obtained from a tertiary hospital in Anshan, Liaoning Province, covering inpatient records from January 2018 to December 2024. Patients were included if they had a primary diagnosis coded as one of the following ICD-10 codes: S22, S32, S42, S52, S62, S72, S82, or S92. Exclusion criteria included a length of stay of less than 1 day, or missing/zero total hospitalization costs. Cost data from 2018 to 2024 were adjusted using annual inflation factors published by the National Bureau of Statistics to account for price changes. Owing to the lack of systematic assessment data on patient frailty, this variable was not included in the present study; however, previous research has demonstrated that frail patients are at higher risk of postoperative complications and incur greater economic burdens (32).

Based on information from the front page of the medical records, we extracted data on patient name, age, sex, length of hospital stay, and total hospitalization cost. Hospitalization costs were further categorized into the following components: general medical service fees, diagnostic fees, therapeutic fees, rehabilitation fees, traditional Chinese medicine (TCM) fees, consumables, and medication costs. Medication costs were further subdivided into Western medicine and traditional Chinese medicine.

**Figure 2:**
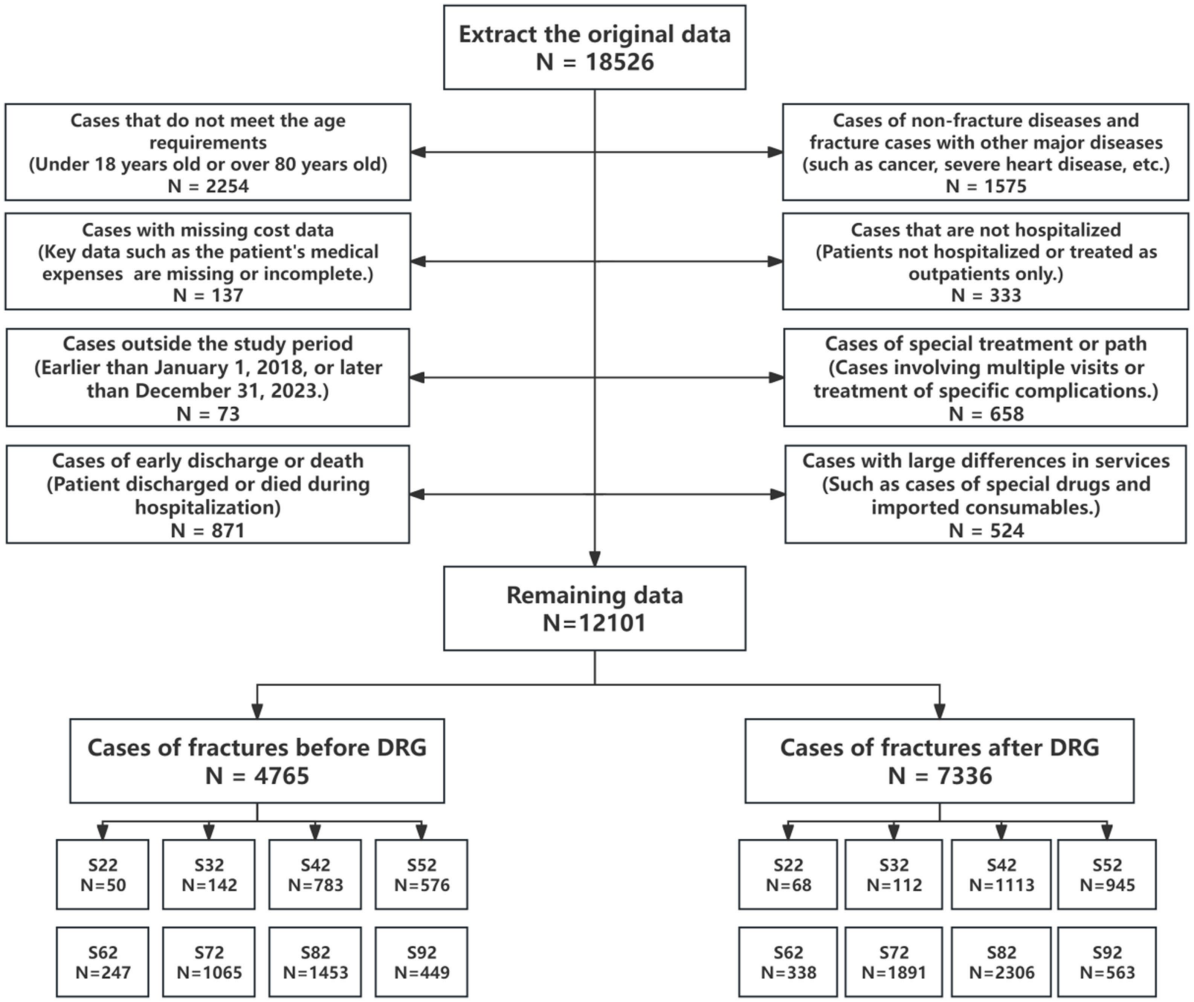
Inclusion and exclusion flowchart.

A total of 12,101 eligible cases were included in the final analysis. The patient selection flowchart is shown in Figure 2.

### 2.2 Research methods

#### 2.2.1 Structural variation analysis

Structural Variation Analysis is a quantitative method used to evaluate and analyze changes in the composition of healthcare expenditures across different periods or policy conditions. In this study, it was employed to identify the variation in the proportion of various expense categories (e.g., diagnostic, therapeutic, rehabilitation, pharmaceutical, and consumables) within the total hospitalization cost of fracture patients before and after the DRG payment reform, as well as their contribution to total cost changes.

This method involves the calculation of three core indicators: value of structure variation (VSV): reflects the absolute change in the proportion of each expense category. Degree of structure variation (DsV): measures the overall extent of change in the cost structure. Contribution rate to structure variation (CRSV): evaluates the relative contribution of each category to the total structural change.

To ensure analytical rigor, normality tests were performed on the distribution of each expense category and the total cost prior to analysis. For normally distributed variables (e.g., diagnostic fees in certain groups), mean and standard deviation were used for descriptive and comparative purposes. For non-normally distributed variables (e.g., drug and rehabilitation costs), the median and interquartile range were used as indicators of central tendency and dispersion. This approach ensured the robustness and interpretability of the findings, especially under conditions of skewed distributions or long-tail effects.

In addition to capturing static shifts in cost composition, this method also facilitates a dynamic understanding of how DRG reforms influenced expenditure patterns, particularly in suppressing unnecessary spending (e.g., pharmaceuticals and consumables) and promoting rational investments (e.g., treatment and rehabilitation services). By integrating structural variation analysis with descriptive statistics, the study provides a comprehensive evaluation of the reform’s micro- and macro-level impacts on healthcare costs, offering both empirical support and theoretical insights for policy optimization.

#### 2.2.2 Interrupted time series analysis

To evaluate the long-term impact of the DRG payment reform on the cost structure of hospitalized fracture patients, this study employed ITS analysis. ITS is a quasi-experimental design suitable for situations where the intervention occurs at a well-defined time point and randomization is not feasible. The methodological approach followed the tutorial by Bernal et al. (33). The model was specified as a segmented regression, aiming to simultaneously estimate changes in level and trend before and after the intervention, with the following form: *Y_t_* = β₀ + β₁*T_t_* + β₂ *X_t_* + β₃ (*T_t_* × *X_t_*) + ϵ*_t_* where *Y_t_* represents the mean cost (or a specific cost category) at time point *t*; *T_t_* is the time elapsed from the start of the observation period to time *t*; *X_t_* is a binary variable indicating the intervention (0 = pre-intervention, 1 = post-intervention); and (*T_t_* × *X_t_*) denotes the change in trend after the intervention. The parameters are interpreted as follows: β₀ represents the baseline level (initial value before the intervention); β_1_ indicates the pre-intervention trend (slope); β_2_ reflects the immediate level change following the intervention; and β_3_ represents the change in trend after the intervention. The results are presented by comparing the temporal patterns of various cost categories (e.g., diagnostic, therapeutic, rehabilitation, pharmaceutical, and consumable costs) before and after DRG implementation, thereby providing a clear visualization of the policy’s actual impact on hospital spending behavior.

#### 2.2.3 Gray relational analysis

Gray relational analysis (GRA) is a statistical method designed for complex multivariate systems under conditions of limited or uncertain information. Its core concept is to assess the “relational degree” among variables in order to determine which factors are most closely associated with the target behavior or outcome.

Unlike traditional correlation analysis, GRA does not rely on assumptions about the data distribution. In this study, GRA was used to examine the association between each expense category (e.g., drug costs, diagnostic fees, therapeutic fees, surgical costs, bed charges, etc.) and the structural changes in total hospitalization costs following DRG reform.

We used the time series of structural variation values (e.g., VSV) as the reference sequence and the structural proportion changes of each expense type across pre- and post-DRG periods as comparison sequences to construct the gray relational model. By calculating the gray relational coefficients and their mean values (i.e., relational degree), we were able to determine which expense categories played a leading role in driving the observed cost structure changes.

### 2.3 Statistical methods

All statistical analyses were performed using SPSS version 26.0. Categorical variables were analyzed using the chi-square test to assess group differences. For continuous variables, independent samples t-tests were used to evaluate differences in group means. For variables that did not follow a normal distribution, descriptive statistics were presented as medians and interquartile ranges. A two-sided *p* < 0.05 was considered statistically significant. The median may not fully capture the overall reduction in costs, whereas the total expenditure could provide a more comprehensive picture. However, this approach may oversimplify the analysis, particularly given the potential confounding effects of inflation, patient frailty, and the COVID-19 pandemic—factors that were not fully controlled in this study. Therefore, the median was chosen as the primary indicator to minimize the influence of extreme cases, while the discussion section highlights the potential value and limitations of total expenditure as an alternative measure, allowing readers to interpret cost changes from multiple perspectives.

## 3 Results

### 3.1 Basic characteristics of hospitalized fracture patients

**Figure 3:**
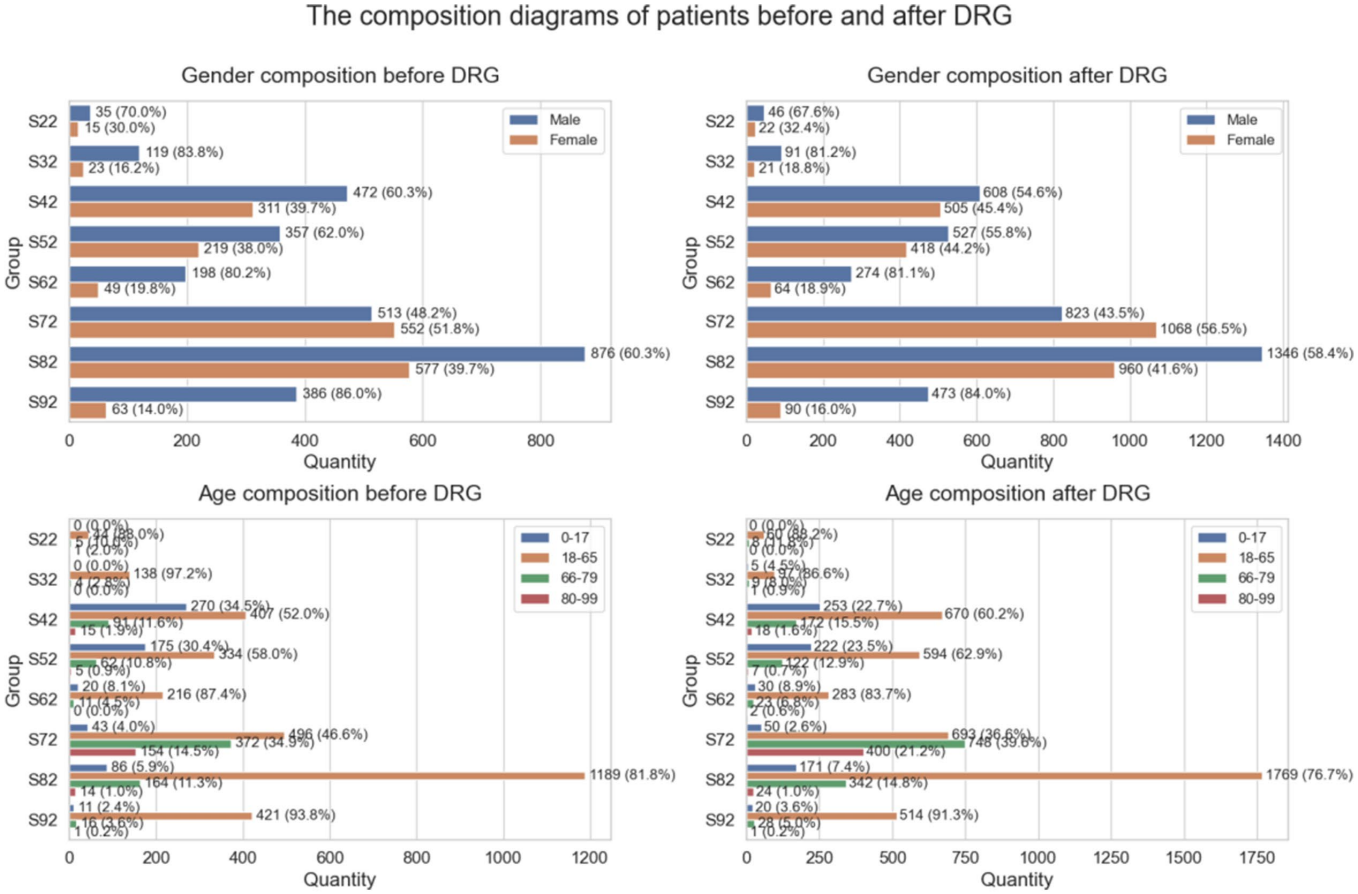
Patient composition before and after DRG implementation (S22–S92). Regarding length of hospital stay, the proportion of patients hospitalized for more than 30 days increased post-DRG, especially in S22, S32, and S42, which may be attributed to more complex treatment plans or higher disease severity. For S72 and S82 patients, there was a marked increase in those with a hospital stay of 15–30 days, suggesting adjustments in therapeutic approaches or recovery timelines.

The composition of patients is shown in Figure 3. According to the clinical data analysis, the proportion of male patients increased across all fracture categories following the implementation of the DRG policy, with a particularly notable rise observed in S72 (femoral fractures) and S82 (lower leg fractures). In terms of age distribution, patients with S22 (rib fractures) and S32 (spinal fractures) were predominantly aged 18–65 years, whereas those with S42, S52, S62, S72, and S82 were mainly older population aged 60 years and above. Notably, the proportion of older population increased after the DRG reform.

Clinical characteristics of the patients are shown in Table 1. Based on the information in Table 1, an increasing trend in the proportion of male patients was observed across multiple disease groups following the implementation of the DRG policy, with particularly pronounced changes in the S72 and S82 groups. In terms of age distribution, the number of patients aged 0–17 years significantly increased in the S32 group, while the number of patients aged 18–65 and 66–79 years markedly increased in the S42 and S52 groups, indicating a relative rise in the proportion of younger and middle-aged patients. Meanwhile, the number of patients aged 0–17 years slightly decreased in the S42 group, reflecting a shift in age structure. All age subgroups in the S72 group showed a significant increase in patient numbers, suggesting a general rise in healthcare utilization across age strata. Regarding the length of hospital stay, after the DRG reform, there was a significant increase in the number of patients in the S72 group hospitalized for 1–14 days and 15–30 days, accompanied by a slight decrease in those hospitalized for over 30 days. In the S82 group, the number of patients hospitalized for both 1–14 days and over 30 days increased significantly. For the S92 group, a marked increase was observed in patients with hospital stays of 15–30 days, indicating possible adjustments in treatment and recovery durations for certain fracture types.

**Table 1:** Clinical characteristics of the patients.

Disease	Time period	Sex		Age (years)				Length of stay (days)		
		Man	Women	0–17	18–65	66–79	80–99	1–14	15–30	>30
S22	Before DRG	35	15	0	44	5	1	2	14	34
	After DRG	46	22	0	60	8	0	5	20	43
	*P*-value	0.964	0.950	NA	1.000	0.996	0.877	0.719	1.000	0.820
S32	Before DRG	119	23	0	138	4	0	11	38	93
	After DRG	91	21	5	97	9	1	19	26	67
	*P*-value	0.858	0.731	0.038*	0.333	0.119	0.905	0.048*	0.651	0.585
S42	Before DRG	472	311	270	407	91	15	383	256	144
	After DRG	608	505	253	670	172	18	535	371	207
	*P*-value	0.080	0.050*	<0.001*	0.010*	0.028*	0.757	0.804	0.834	0.96
S52	Before DRG	357	219	175	334	62	5	322	145	109
	After DRG	527	418	222	594	122	7	534	234	177
	*P*-value	0.093	0.056	0.009*	0.199	0.265	1.000	0.896	0.914	0.981
S62	Before DRG	198	49	20	216	11	0	147	53	47
	After DRG	274	64	30	283	23	2	224	58	56
	*P*-value	0.932	0.877	0.859	0.606	0.317	0.622	0.279	0.263	0.536
S72	Before DRG	513	552	43	496	372	154	360	425	280
	After DRG	823	1,068	50	693	748	400	726	896	269
	*P*-value	0.054	0.075	0.051	<0.001*	0.0389*	<0.001*	0.038*	0.002*	<0.001*
S82	Before DRG	876	577	86	1,189	164	14	469	510	474
	After DRG	1,346	960	171	1769	342	24	829	820	657
	*P*-value	0.419	0.349	0.096	0.047*	0.004*	0.950	0.051	0.829	0.019*
S92	Before DRG	386	63	11	421	16	1	179	133	137
	After DRG	473	90	20	514	28	1	250	125	188
	*P*-value	0.722	0.464	0.413	0.654	0.356	1.000	0.256	0.018*	0.429

*p < 0.05, statistically significant.

### 3.2 Impact of DRG reform on cost structure

**Figure 4:**
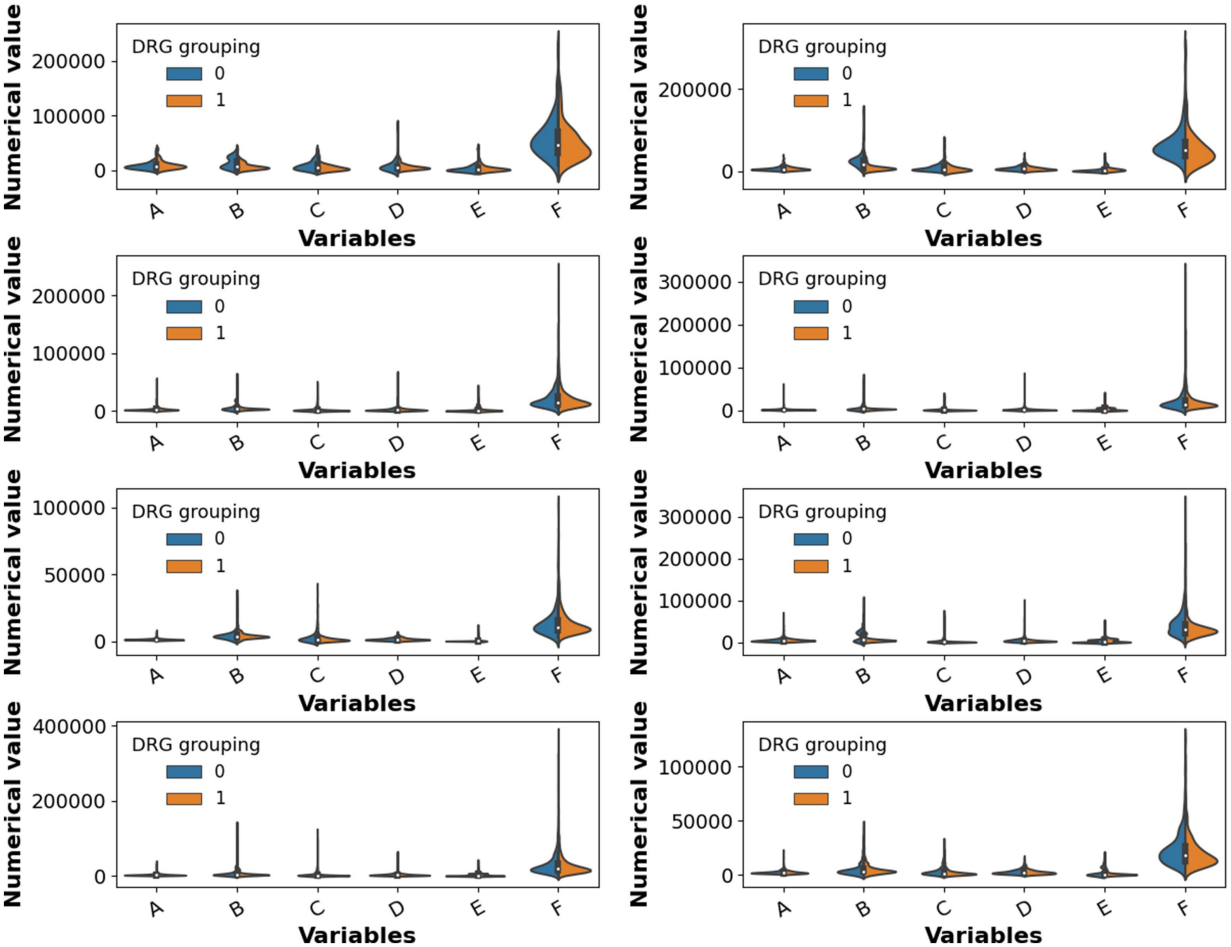
Medical expenditure statistics for S22–S92 before and after DRG implementation. A: Diagnostic services, B: Therapeutic services, C: Rehabilitation services, D: Medication costs, E: Consumables, and F: Total hospitalization cost.

The disease-specific medical expenditures before and after DRG implementation are summarized in Figure 4. Before the reform, the median diagnostic cost was 302.26 CNY with a standard deviation (SD) of 303.88 CNY; after the reform, the median decreased to 273.72 CNY with a reduced SD of 264.96 CNY, indicating a slight reduction in costs and a more concentrated distribution. The median therapeutic cost increased from 1,015.38 CNY (SD: 1,379.04 CNY) before the reform to 1,200.91 CNY (SD: 1,622.64 CNY) after the reform, reflecting a general rise in treatment costs and greater variability. Rehabilitation costs remained relatively low in both periods (median: 0.00 CNY before vs. 47.14 CNY after), but the SD increased from 162.89 CNY to 263.90 CNY, suggesting a substantial increase in rehabilitation interventions post-reform, with a right-skewed distribution emerging due to selective patient uptake. Medication costs dropped significantly, with the median decreasing from 3,416.06 CNY to 2,796.74 CNY, and SD declining from 2,399.26 CNY to 1,968.34 CNY. Consumable costs showed a slight increase in median from 7,358.12 CNY to 7,465.64 CNY, accompanied by greater variability (SD: from 5,492.94 CNY to 6,117.88 CNY), indicating relatively stable central tendency but increased dispersion. The total hospitalization cost decreased marginally from a median of 19,388.31 CNY to 18,609.57 CNY, while the SD slightly increased from 8,120.89 CNY to 8,269.52 CNY. Overall, DRG implementation resulted in a notable decline in medication expenditures and a moderate reduction in diagnostic costs, while therapeutic and rehabilitation expenditures showed upward trends. Although the total cost decreased slightly, the variability remained, suggesting considerable individual differences. These findings imply that further refinement of the DRG payment system—such as incorporating disease severity stratification—remains necessary for more precise cost control.

### 3.3 Structural variation analysis results

**Figure 5:**
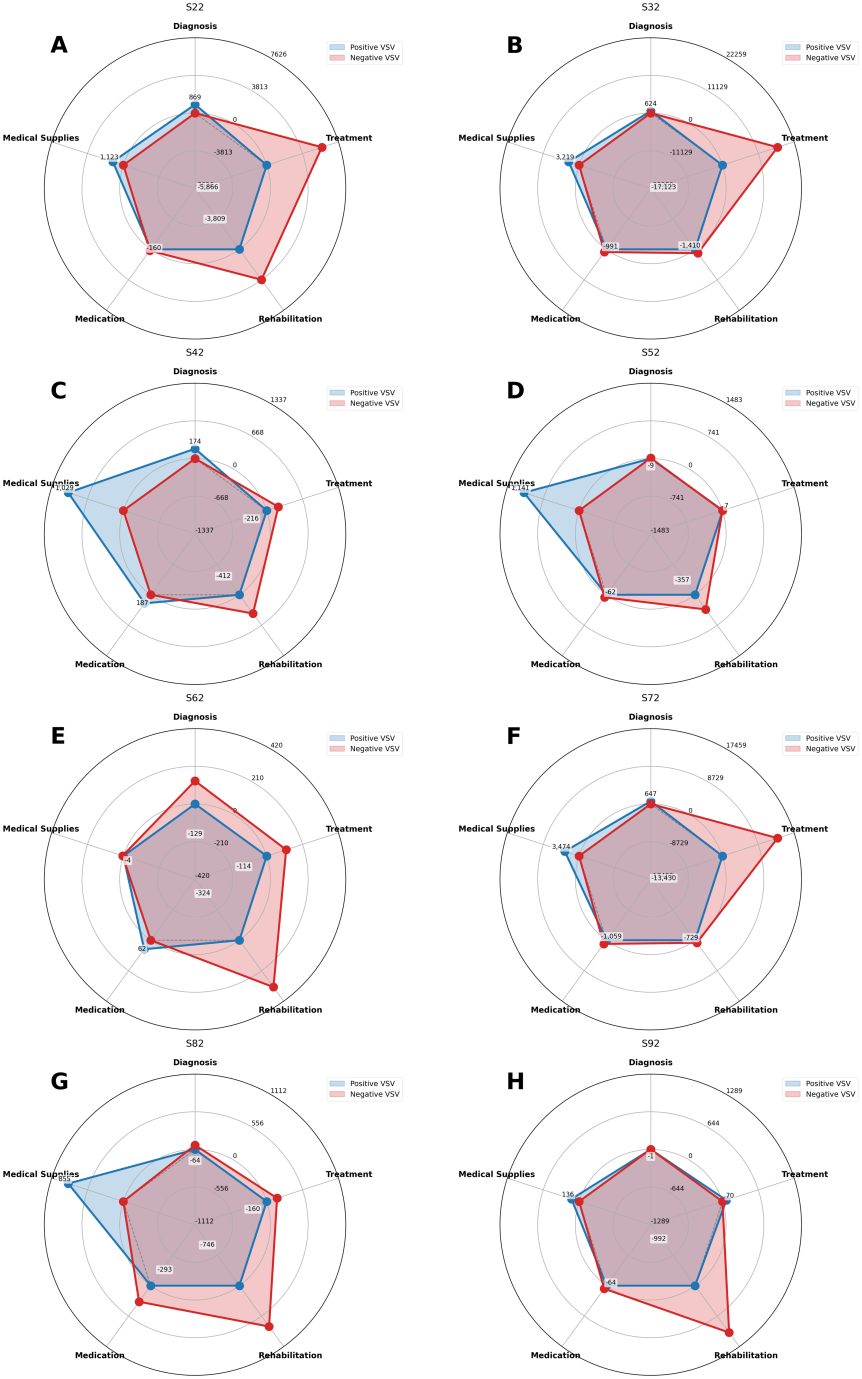
Radar chart of VSV for related cost categories across fracture types S22 to S92 **(A)**: S22 VSV analysis, **(B)**: S32 VSV analysis, **(C)**: S42 VSV analysis, **(D)**: S42 VSV analysis, **(E)**: S52 VSV analysis, **(F)**: S62 VSV analysis, **(G)**: S72 VSV analysis, **(H)**: S82 VSV analysis.

In the cost structures of different fracture types (S22, S32, S42, S52, S62, S72, S82, S92), the VSV reflected the absolute changes across various categories. Diagnostic costs ranged from −128.84 CNY to 868.92 CNY, with an increase of 868.92 CNY in S22 and a decrease of 128.84 CNY in S62; the corresponding changes for other fracture types were 623.70 CNY (S32), 174.00 CNY (S42), −9.20 CNY (S52), 646.76 CNY (S72), −63.55 CNY (S82), and −1.36 CNY (S92). Therapeutic costs showed the largest variation, ranging from −17,123.05 CNY (S32) to 70.00 CNY (S92), indicating substantial fluctuations before and after DRG implementation. Rehabilitation costs decreased across all fracture types, ranging from −991.80 CNY (S92) to −3,809.00 CNY (S22), with changes of −1,410.05 CNY (S32), −412.20 CNY (S42), −356.73 CNY (S52), −323.60 CNY (S62), −729.40 CNY (S72), and −745.60 CNY (S82). Pharmaceutical costs ranged from −1,058.80 CNY (S72) to 187.22 CNY (S42); the corresponding values for other fractures were −160.44 CNY (S22), −990.90 CNY (S32), −62.07 CNY (S52), 62.36 CNY (S62), −293.15 CNY (S82), and −64.05 CNY (S92). Consumables costs exhibited a marked upward trend, ranging from 1,029.06 CNY (S42) to 3,474.02 CNY (S72), with other values being 1,123.01 CNY (S22), 3,218.84 CNY (S32), 1,141.44 CNY (S52), −4.28 CNY (S62), 855.39 CNY (S82), and 136.34 CNY (S92). Overall, therapeutic costs experienced the most substantial decline, while consumables costs demonstrated a clear upward trend (VSV values for each fracture type and cost category are shown in Figure 5).

The DsV of each cost category reflected the variation in their proportions of total expenditure. Diagnostic DsV ranged from −29.12% (S62) to 79.69% (S32), with values of 1.95% (S22), −12.11% (S42), −32.51% (S52), −8.66% (S72), −28.27% (S82), and −9.02% (S92). Therapeutic costs exhibited the most pronounced changes, ranging from −77.41% (S72) to −7.02% (S92), corresponding to absolute reductions between −14,508.43 CNY (S72) and −228.67 CNY (S92), indicating that the share of therapeutic costs in total expenditure decreased the most substantially. Rehabilitation DsV ranged from −57.28% (S52) to 16.73% (S32), corresponding to changes from −1,065.41 CNY to 900.01 CNY. Pharmaceutical DsV varied between −39.34% (S72) and 31.05% (S32), with corresponding absolute changes from −1,735.87 CNY to 1,793.04 CNY. Consumables showed the widest DsV variation, from 111.0% (S92) to 885.22% (S52), with absolute changes ranging from 115.64 CNY to 5,236.68 CNY, demonstrating considerable fluctuations in their share of total costs.

**Figure 6:**
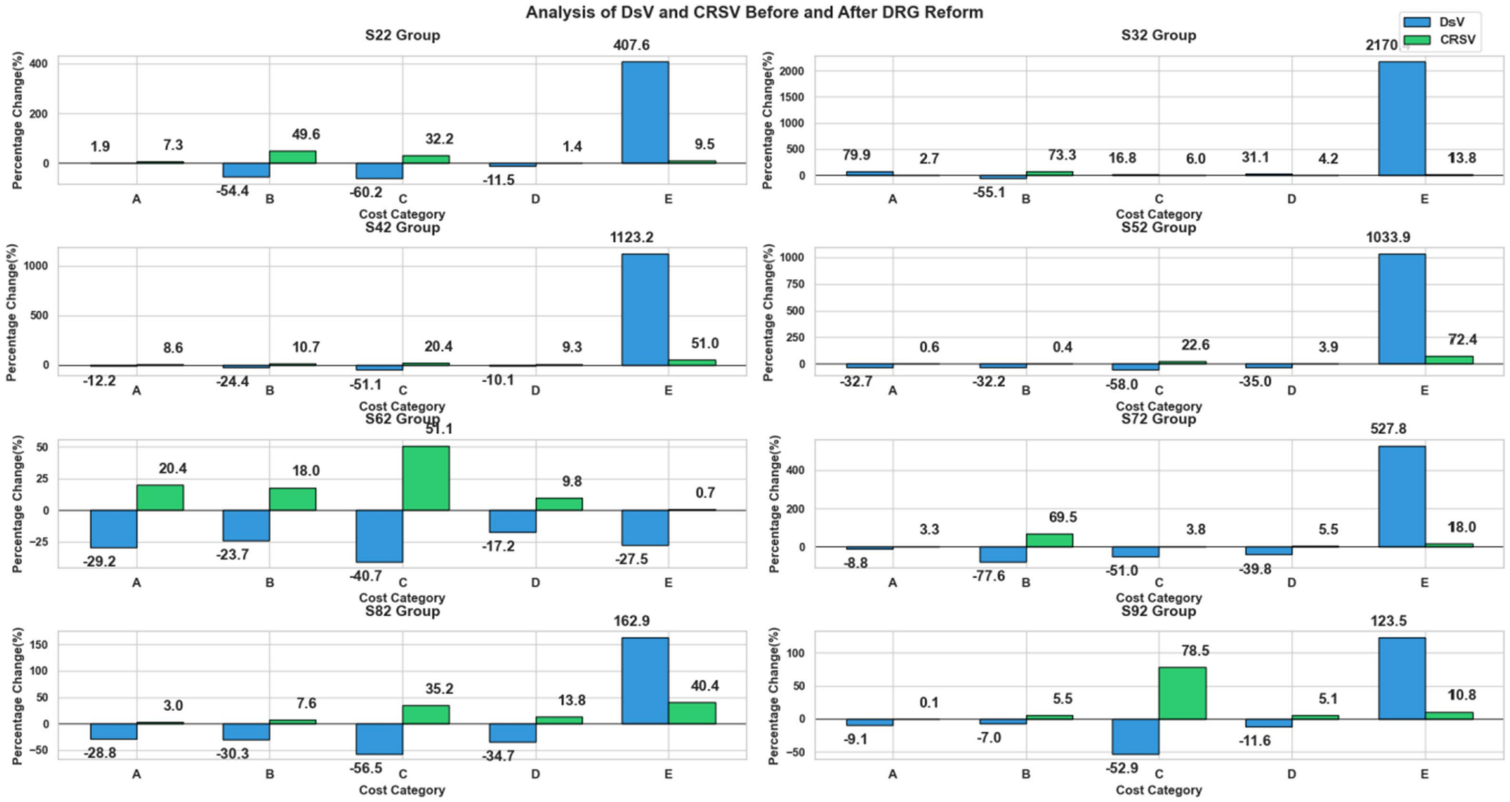
DsV and CRSV results for related cost categories across S22–S92 groups. A: Diagnostic costs, B: Treatment costs, C: Rehabilitation costs, D: Medication costs, and E: Consumable costs.

Substantial heterogeneity was also observed in the CRSV across cost categories. Therapeutic costs contributed the most to overall structural variation in most fracture types, reaching as high as 73.28% in S32 and 69.45% in S72, highlighting their dominant role as drivers of structural change. Rehabilitation costs showed moderate contributions, ranging from 32.20% in S22 to 78.49% in S92. Diagnostic costs contributed 0.11% (S92) to 20.35% (S62), while pharmaceutical costs showed relatively low contributions, with 9.27% in S42 and 3.94% in S52. Consumables displayed marked variability, with contributions ranging from only 0.68% in S62 to as high as 72.41% in S52, suggesting that while consumables had substantial impacts in certain fracture types, their contribution was less consistent compared with therapeutic costs. The DsV and CRSV results for related cost categories across groups S22 to S92 are shown in Figure 6.

### 3.4 Interrupted time series analysis results

**Figure 7:**
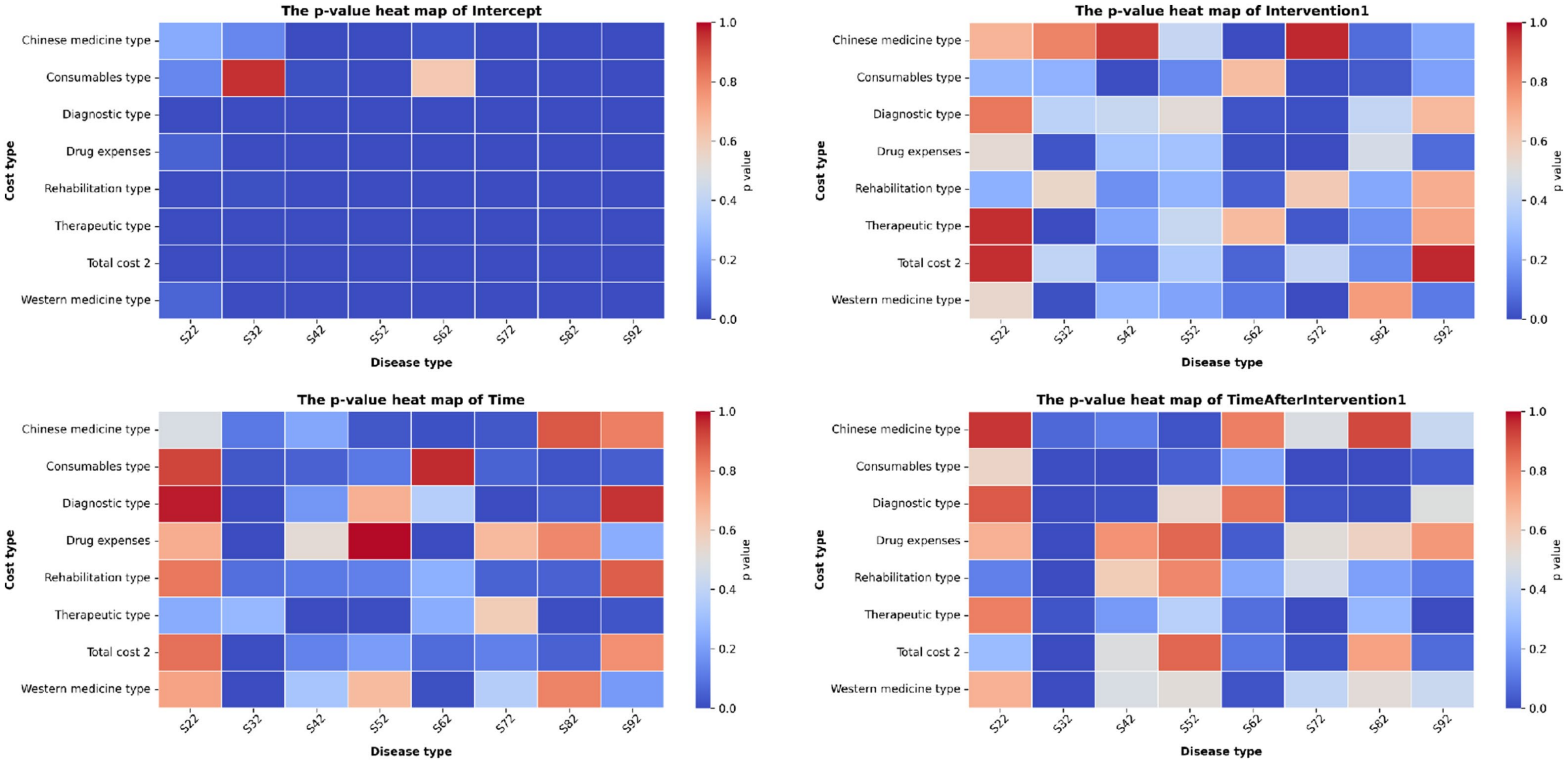
Heatmap of interrupted time series analysis results.

ITS results for cost categories (S22–S92) are shown in Table 2 and ITS results, shown in Figure 7, indicate significant differences (*p* < 0.05) in the impact of the intervention across different cost categories and fracture types. Regarding total costs, the pre-DRG trends across fracture types showed a divergent pattern: while most types exhibited declining trends, some, notably S32 fractures, demonstrated a significant upward trajectory (β₁ = 1247.93, *p* < 0.001). Post-implementation, a systematic shift occurred: previously declining trends moderated, while previously rising trends were reversed. This reversal was most pronounced in S32 fractures, where total costs shifted from pre-policy increase to a rapid decrease (β₃ = −2467.0, *p* < 0.001). For diagnostic costs, a general downward trend was observed pre-policy (e.g., S82: β₁ = −24.92, *p* = 0.032), with the exception of S32 fractures, which showed a significant increase (β₁ = 171.87, *p* = 0.002). Post-policy, the trends for diagnostic costs largely reversed direction across types. S32 fractures showed the most marked decline (β₃ = −227.16, *p* < 0.001), whereas S72 fractures exhibited the strongest increasing trend (β₃ = 52.86, *p* = 0.017). Treatment costs generally displayed declining or weakly rising trends before the policy. After implementation, the declining trends intensified, with the rate of decrease accelerating for the vast majority of fracture types. Typically, S92 fractures saw their treatment costs reverse from a slow pre-policy increase (β₁ = 64.68, *p* = 0.023) to a rapid decrease (β₃ = −157.59, *p* < 0.001). Similarly, the declining trend for S72 fractures significantly accelerated (β₃ = −258.9, *p* < 0.001). Medication costs mostly showed slight upward trends pre-policy, most prominently in S32 fractures (β₁ = 259.43, *p* < 0.001). Post-policy, these generally shifted to downward trends, with S32 fractures again showing the largest decrease (β₃ = −355.1, *p* < 0.001). Further analysis revealed that Western medicine costs were the primary driver of this change. The trends for Western and TCM costs before and after the policy were largely consistent with the overall medication cost changes. However, for some fracture types, TCM cost trends moved inversely to those of Western medicine. Consumables costs were predominantly increasing before the policy, with only S92 fractures showing a decrease (β₁ = −50.08, *p* = 0.042). Post-policy, trends diverged significantly: the increasing trends for types like S32, S72, and S82 were effectively controlled and reversed to decreases (e.g., S72: β₃ = −114.1, *p* = 0.002). In contrast, the upward trend for S42 fractures intensified sharply (β₃ = 1578.62, *p* < 0.001). Rehabilitation costs were mostly declining pre-policy. After the policy, all fracture types switched to decreasing trends for rehabilitation costs, with most types showing a strengthened decline or a reversal from increase to decrease. The control effect was most evident in S32 fractures, where rehabilitation costs reversed from a pre-policy increase to a sharp decrease (β₃ = −483.58, *p* < 0.001).

**Table 2:** Interrupted time series results for cost categories (S22–S92).

Disease	Category	Β_0_	Β_1_	Β_2_	Β_3_
S22	Total cost	60217.04	142.98	−810.08	−1019.25
	*P*-value	<0.001*	0.841	0.960	0.289
	Diagnostic	8760.73	−3.73	744.84	28.86
	*P*-value	<0.001*	0.980	0.826	0.887
	Therapeutic	17527.14	−202.56	190.63	−55.21
	*P*-value	<0.001*	0.241	0.961	0.811
	Consumables	2751.47	11.1	2,918	−91.62
	*P*-value	0.140	0.925	0.272	0.563
	Drug expenses	7780.25	96.92	−3530.96	−135.66
	*P*-value	0.052	0.698	0.531	0.687
	Western medicine	7551.87	88.31	−3417.11	−136.75
	*P*-value	0.059	0.724	0.544	0.684
	Traditional Chinese medicine	228.38	8.62	−113.86	1.09
	*P*-value	0.238	0.482	0.679	0.947
	Rehabilitation	8870.06	−28.4	3346.11	−273.1
	*P*-value	<0.001*	0.826	0.254	0.122
S32	Total cost	47357.65	1247.93	−9022.77	−2,467
	*P*-value	<0.001*	<0.001*	0.402	<0.001*
	Diagnostic	3539.57	171.87	−1142.18	−227.16
	*P*-value	<0.001*	0.002*	0.387	0.001*
	Therapeutic	23523.88	162.92	−12987.91	−441.62
	*P*-value	<0.001*	0.278	<0.001*	0.020*
	Consumables	−66.06	151.29	1831.45	−228.84
	*P*-value	0.955	0.026*	0.260	0.007*
	Drug expenses	3614.04	259.43	−3444.08	−355.1
	*P*-value	0.001*	<0.001*	0.021*	<0.001*
	Western medicine	3137.54	229.52	−3334.56	−312.86
	*P*-value	<0.001*	<0.001*	0.009*	<0.001*
	Traditional Chinese medicine	476.51	29.91	−109.52	−42.24
	*P*-value	0.138	0.104	0.804	0.066
	Rehabilitation	5103.57	197.51	1597.24	−483.58
	*P*-value	0.011*	0.080	0.554	<0.001*
S42	Total cost	25708.33	−144.29	4108.9	−79.45
	*P*-value	<0.001*	0.129	0.083	0.494
	Diagnostic	2828.71	−15.4	225.76	34.75
	*P*-value	<0.001*	0.177	0.424	0.015*
	Therapeutic	9,170	−120.57	871.38	46.81
	*P*-value	<0.001*	<0.001*	0.224	0.186
	Consumables	1011.84	43.49	1578.62	−121.81
	*P*-value	0.011*	0.048*	0.005*	<0.001*
	Drug expenses	2405.72	8.46	−332.47	−4.83
	*P*-value	<0.001*	0.525	0.317	0.768
	Western medicine	1993.46	11.9	−337.96	−10.41
	*P*-value	<0.001*	0.325	0.262	0.483
	Traditional Chinese medicine	412.27	−3.45	5.49	5.58
	*P*-value	<0.001*	0.223	0.938	0.11
	Rehabilitation	2947.53	−30.69	660.74	−12.27
	*P*-value	<0.001*	0.107	0.163	0.597
S52	Total cost	24168.19	−135.65	2435.01	−22.56
	*P*-value	<0.001*	0.197	0.350	0.861
	Diagnostic	2648.8	−5.65	−226.49	10.65
	*P*-value	<0.001*	0.691	0.523	0.543
	Therapeutic	6975.59	−83.59	574.35	31.26
	*P*-value	<0.001*	0.005*	0.423	0.377
	Consumables	1658.93	37.79	847.95	−57.98
	*P*-value	<0.001*	0.105	0.143	0.044*
	Drug expenses	2571.07	0.09	−440.56	−3.75
	*P*-value	<0.001*	0.996	0.315	0.862
	Western medicine	2035.77	7.39	−505.24	−13.21
	*P*-value	<0.001*	0.653	0.218	0.513
	Traditional Chinese medicine	535.3	−7.3	64.68	9.46
	*P*-value	<0.001*	0.024*	0.414	0.018*
	Rehabilitation	3133.91	−37.13	666.64	−7.8
	*P*-value	<0.001*	0.122	0.263	0.79
S62	Total cost	11930.81	159.8	−4212.62	−180.3
	*P*-value	<0.001*	0.073	0.058	0.099
	Diagnostic	1362.47	4.22	−282.19	1.24
	*P*-value	<0.001*	0.368	0.018*	0.829
	Therapeutic	3853.34	30.92	−295.19	−57.45
	*P*-value	<0.001*	0.246	0.655	0.081
	Consumables	102.11	0.53	125.9	17.15
	*P*-value	0.612	0.962	0.65	0.213
	Drug expenses	1045.77	29.12	−590.42	−23.09
	*P*-value	<0.001*	0.002*	0.009*	0.036*
	Western medicine	895.59	20.11	−303.18	−22.04
	*P*-value	<0.001*	0.010*	0.109	0.019*
	Traditional Chinese medicine	150.18	9.01	−287.24	−1.05
	*P*-value	0.022*	0.014*	0.001*	0.811
	Rehabilitation	2239.42	36.20	−1590.82	−47.37
	*P*-value	<0.001*	0.254	0.0463*	0.225
S72	Total cost	48590.46	−151.99	−2004.7	−283.3
	*P*-value	<0.001*	0.124	0.412	0.021*
	Diagnostic	6579.32	−58.1	1112.07	52.86
	*P*-value	<0.001*	0.002*	0.014*	0.017*
	Therapeutic	18136.15	−28.51	−2990.47	−258.9
	*P*-value	<0.001*	0.596	0.028*	<0.001*
	Consumables	2680.93	55.86	1930.05	−114.1
	*P*-value	<0.001*	0.052	0.008*	0.002*
	Drug expenses	5303.6	7.71	−1430.96	−14.23
	*P*-value	<0.001*	0.663	0.002*	0.513
	Western medicine	4553.79	14.94	−1427.82	−16.97
	*P*-value	<0.001*	0.366	<0.001*	0.403
	Traditional Chinese medicine	749.81	−7.22	−3.14	2.74
	*P*-value	<0.001*	0.026*	0.968	0.485
	Rehabilitation	4508.24	−35.77	−232.1	−16.63
	*P*-value	<0.001*	0.052	0.607	0.456
S82	Total cost	34,466	−204.21	3728.7	−43.33
	*P*-value	<0.001*	0.049*	0.146	0.730
	Diagnostic	3751.54	−24.92	235.72	37.5
	*P*-value	<0.001*	0.032*	0.408	0.010*
	Therapeutic	8885.72	−87.93	1046.83	40.11
	*P*-value	<0.001*	0.005*	0.164	0.279
	Consumables	2126.8	40.98	887.18	−92.42
	*P*-value	<0.001*	0.016	0.035*	<0.001*
	Drug expenses	3853.39	−3.37	−232.91	−8.86
	*P*-value	<0.001*	0.792	0.465	0.573
	Western medicine	3197.94	−2.94	−95.22	−9.25
	*P*-value	<0.001*	0.801	0.743	0.52
	Traditional Chinese medicine	655.45	−0.43	−137.7	0.39
	*P*-value	<0.001*	0.889	0.077	0.917
	Rehabilitation	5186.61	−47.72	718.26	−37.16
	*P*-value	<0.001*	0.048*	0.227	0.206
S92	Total cost	23579.45	25.09	−87.31	−194.79
	*P*-value	<0.001*	0.772	0.968	0.071
	Diagnostic	2362.91	−0.61	112.75	8.59
	*P*-value	<0.001*	0.953	0.661	0.499
	Therapeutic	4553.84	64.68	249.57	−157.59
	*P*-value	<0.001*	0.023*	0.720	<0.001*
	Consumables	3403.61	−50.08	−766.85	62.99
	*P*-value	<0.001*	0.042*	0.207	0.038*
	Drug expenses	2634.57	15.11	−587.24	−5.05
	*P*-value	<0.001*	0.245	0.072	0.751
	Western medicine	2103.85	13.85	−428.21	−10.19
	*P*-value	<0.001*	0.191	0.106	0.432
	Traditional Chinese medicine	530.72	1.25	−159.03	5.14
	*P*-value	<0.001*	0.810	0.223	0.424
	Rehabilitation	3783.09	−3.62	−228.86	−46.62
	*P*-value	<0.001*	0.879	0.698	0.113

β₀: baseline level (initial value before the intervention); β₁: pre-intervention trend (slope); β₂: immediate change in level following the intervention and β₃: change in trend after the intervention.

*p < 0.05, statistically significant.

### 3.5 Gray relational analysis results

Gray relational analysis revealed that the correlations between cost categories and total hospitalization expenses remained generally high across all fracture types (average range: 0.7–0.9) before and after DRG implementation, though distinct patterns of variation were observed. In S22 fractures, diagnostic and pharmaceutical costs increased from 0.7396 to 0.7560 and from 0.6879 to 0.7375, respectively, with Western medicine also rising from 0.6862 to 0.7366, indicating a general increase in diagnostic- and drug-related relevance. In contrast, treatment and rehabilitation decreased from 0.7679 and 0.7659 to 0.7285 and 0.7276. In S32 fractures, material costs increased markedly from 0.7281 to 0.8067, while treatment, Western medicine, overall medication, and diagnostic expenses remained high (0.79–0.80), suggesting a relatively stable upward trend. S42 fractures showed consistent increases across all categories, with Western medicine, overall medication, material, rehabilitation, diagnostic, and traditional Chinese medicine rising from 0.82 to 0.89–0.90, indicating highly concentrated cost structures. S52 fractures exhibited a uniform decline. Treatment, material, and rehabilitation costs dropped from 0.9060, 0.9149, and 0.9146 to 0.7968, 0.7867, and 0.7747, respectively. Western medicine, overall medication, diagnostic, and traditional medicine costs also fell into the 0.77–0.78 range, reflecting reduced coupling with total costs. In S62 fractures, all categories declined, with treatment falling from 0.8766 to 0.7949, Western and overall medication from 0.8478 and 0.8497 to 0.7600 and 0.7573, and material and traditional medicine to 0.7537 and 0.7506. Diagnostic costs decreased from 0.8462 to 0.7338, with overall reductions ranging 0.06–0.11. S72 fractures showed relative stability: material, diagnostic, traditional, and medication costs remained between 0.81–0.83; treatment declined slightly from 0.8636 to 0.8128; and rehabilitation stabilized around 0.82, reflecting minimal fluctuation. In S82 fractures, nearly all categories showed modest increases. Material rose from 0.8871 to 0.8946, rehabilitation from 0.8856 to 0.8937, and medication and Western medicine from 0.8772 and 0.8766 to 0.8932 and 0.8917. Traditional medicine and diagnostic categories increased from 0.8805 and 0.8808 to 0.8870 and 0.8853, respectively, while treatment rose slightly to 0.8645. S92 fractures displayed broad-based increases: overall medication and Western medicine rose from 0.7666 and 0.7633 to 0.8453 and 0.8422; material from 0.7741 to 0.8396; diagnostic and rehabilitation to 0.8395; traditional medicine to 0.8386; and treatment from 0.7750 to 0.8335, representing the most evenly distributed gains among all groups (see Table 3).

**Table 3:** Gray relational analysis results for cost categories (S22–S92) before and after DRG implementation.

Disease	Expense types	Correlation degree before DRG	95%CI	Correlation degree after DRG	95%CI
S22	Diagnosis	0.7396	[0.6565, 0.7924]	0.7560	[0.6905, 0.7938]
	Treatment	0.7679	[0.6900, 0.7971]	0.7285	[0.6651, 0.8028]
	Rehabilitation	0.7659	[0.6709, 0.8157]	0.7276	[0.6804, 0.7935]
	Consumables	0.7494	[0.6643, 0.7901]	0.7153	[0.6709, 0.7780]
	Medication	0.6879	[0.6465, 0.7733]	0.7375	[0.6909, 0.7923]
	Western medicine	0.6862	[0.6474, 0.7705]	0.7366	[0.6877, 0.7934]
	Traditional Chinese medicine	0.7473	[0.6747, 0.7963]	0.7239	[0.6822, 0.7859]
S32	Diagnosis	0.7371	[0.6532, 0.7931]	0.7929	[0.6452, 0.8164]
	Treatment	0.8005	[0.6888, 0.8401]	0.7984	[0.6580, 0.8232]
	Rehabilitation	0.7387	[0.6727, 0.8019]	0.7662	[0.6483, 0.7962]
	Consumables	0.7281	[0.6519, 0.7768]	0.8067	[0.6523, 0.8293]
	Medication	0.7396	[0.6466, 0.7882]	0.7973	[0.6517, 0.8218]
	Western medicine	0.7424	[0.6480, 0.7940]	0.7977	[0.6550, 0.8206]
	Traditional Chinese medicine	0.744	[0.6590, 0.7941]	0.7902	[0.6568, 0.8164]
S42	Diagnosis	0.8198	[0.8096, 0.8288]	0.8983	[0.8501, 0.9043]
	Treatment	0.8197	[0.8118, 0.8316]	0.8903	[0.8506, 0.8990]
	Rehabilitation	0.8192	[0.8070, 0.8304]	0.8988	[0.8434, 0.9052]
	Consumables	0.8347	[0.8159, 0.8429]	0.9011	[0.8699, 0.9077]
	Medication	0.8233	[0.8131, 0.8327]	0.9017	[0.8572, 0.9076]
	Western Medicine	0.8226	[0.8110, 0.8318]	0.9019	[0.8566, 0.9080]
	Traditional Chinese medicine	0.8254	[0.8097, 0.8340]	0.8977	[0.8579, 0.9039]
S52	Diagnosis	0.8972	[0.8090, 0.9032]	0.774	[0.7503, 0.8072]
	Treatment	0.906	[0.8233, 0.9141]	0.7968	[0.7676, 0.8048]
	Rehabilitation	0.9146	[0.8097, 0.9198]	0.7747	[0.7466, 0.8068]
	Consumables	0.9149	[0.8376, 0.9202]	0.7867	[0.7713, 0.8237]
	Medication	0.8959	[0.8264, 0.9023]	0.7859	[0.7590, 0.8172]
	Western medicine	0.8942	[0.8332, 0.9001]	0.7822	[0.7593, 0.8168]
	Traditional Chinese medicine	0.9091	[0.8316, 0.9141]	0.7740	[0.7474, 0.8068]
S62	Diagnosis	0.8462	[0.7755, 0.8617]	0.7338	[0.7194, 0.7618]
	Treatment	0.8766	[0.8056, 0.8900]	0.7949	[0.7676, 0.8099]
	Rehabilitation	0.8123	[0.7815, 0.8314]	0.7735	[0.7307, 0.8096]
	Consumables	0.8407	[0.7797, 0.8587]	0.7537	[0.7394, 0.7793]
	Medication	0.8497	[0.7801, 0.8710]	0.7573	[0.7419, 0.8060]
	Western medicine	0.8478	[0.7875, 0.8697]	0.7600	[0.7399, 0.8035]
	Traditional Chinese medicine	0.8420	[0.7784, 0.8611]	0.7506	[0.7297, 0.7940]
S72	Diagnosis	0.8025	[0.7533, 0.8116]	0.8235	[0.7935, 0.8282]
	Treatment	0.8636	[0.7717, 0.8735]	0.8128	[0.7789, 0.8231]
	Rehabilitation	0.8273	[0.7390, 0.8332]	0.8131	[0.7570, 0.8199]
	Consumables	0.8273	[0.7635, 0.8336]	0.8368	[0.8013, 0.8447]
	Medication	0.7887	[0.7551, 0.7962]	0.818	[0.7885, 0.8252]
	Western medicine	0.7891	[0.7581, 0.7973]	0.8156	[0.7760, 0.8228]
	Traditional Chinese medicine	0.8195	[0.7501, 0.8262]	0.8221	[0.7828, 0.8290]
S82	Diagnosis	0.8808	[0.8266, 0.8847]	0.8853	[0.8266, 0.8897]
	Treatment	0.8574	[0.8007, 0.8643]	0.8645	[0.8108, 0.8693]
	Rehabilitation	0.8856	[0.8220, 0.8927]	0.8937	[0.8377, 0.8981]
	Consumables	0.8871	[0.8379, 0.8932]	0.8946	[0.8451, 0.8998]
	Medication	0.8772	[0.8376, 0.8830]	0.8932	[0.8381, 0.8974]
	Western medicine	0.8766	[0.8387, 0.8823]	0.8917	[0.8360, 0.8957]
	Traditional Chinese medicine	0.8805	[0.8321, 0.8862]	0.887	[0.8297, 0.8918]
S92	Diagnosis	0.7525	[0.7266, 0.7762]	0.8395	[0.7846, 0.8486]
	Treatment	0.775	[0.7250, 0.7973]	0.8335	[0.7724, 0.8432]
	Rehabilitation	0.7726	[0.7338, 0.7878]	0.8395	[0.7766, 0.8480]
	Consumables	0.7741	[0.7509, 0.7950]	0.8396	[0.7801, 0.8496]
	Medication	0.7666	[0.7473, 0.7899]	0.8453	[0.8067, 0.8547]
	Western medicine	0.7633	[0.7417, 0.7895]	0.8422	[0.7998, 0.8514]
	Traditional Chinese medicine	0.7635	[0.7438, 0.7854]	0.8386	[0.7908, 0.8495]

## 4 Discussion

With the accelerating aging process in China and the continuous rise in trauma incidence, the economic burden of fracture treatment has become a critical issue in public health. As a key measure to control medical insurance costs, the implementation of the DRG payment reform has shown significant differences in its effects across various types of fracture patients. This study systematically evaluated the impact of the DRG reform on the cost structure of eight common fracture groups through structural variation analysis, interrupted time series, and gray relational analysis, revealing three notable phenomena: cost substitution effects characterized by a general decline in treatment costs alongside differentiated changes in consumables expenses; advantages of TCM and age-related differences, reflecting varied treatment demands among different age groups. These findings provide empirical evidence for optimizing trauma payment policies. This phenomenon can be partially explained by the inherent mechanisms of DRG-based payment. Because DRGs emphasize “same price for the same disease,” hospitals facing fixed payments often proactively adjust resource allocation to avoid losses from high-cost cases. Evidence from international studies supports this effect; for example, research in Switzerland has shown that DRGs encourage hospitals to focus more on efficiency and process management, but may also lead to tendencies such as selective admission or early discharge (34).

Following DRG implementation, the proportion of male patients significantly increased, especially in S72 (femoral fractures) and S82 (lower leg fractures) groups, possibly indicating gender-specific healthcare-seeking behaviors under the new payment system. The proportion of older population (≥60 years) rose across S42 to S92 groups, suggesting that DRG may have influenced patient admission standards and thus patient demographics. Notably, the proportion of patients with hospital stays exceeding 30 days increased in S22, S32, and S42 groups, while the 15–30 day hospitalization rate rose in S72 and S82 groups. Such variations may result from differences in fracture site complexity or reflect strategic responses by medical institutions to DRG payment standards (35, 36). Moreover, the observed disparities by sex and age may also reflect broader social factors. Previous studies have indicated that male labor-force populations are more likely to opt for hospitalization following trauma, whereas female and older population are often constrained by family support and rehabilitation resources (37–39). The DRG reform appears to have amplified these differences, suggesting that future payment system designs should not only consider disease-specific clinical characteristics but also account for factors such as sex, age, and social support networks to enhance policy equity and generalizability. The most significant cost structure adjustments were observed in medication expenditures, with median costs decreasing by 12.1% overall after DRG reform (up to 39.8% decrease in the S72 group). In contrast, treatment and consumable costs exhibited opposite trends: treatment costs generally declined (S72 group DsV = −77.6%), indicating that DRG’s fixed payment may suppress high-intensity therapeutic interventions, while consumable costs increased sharply (S32 + 2170.4%, S72 + 527.8%), suggesting a “consumable substitution effect” whereby institutions compensate for lost revenues by increasing consumable usage. Although rehabilitation costs remained low (median increased from 0 to 47.1 yuan), there were significant differences between groups (decreasing in S72 vs. increasing in S82), indicating that DRG coverage of rehabilitation services remains insufficient. By contrast, foreign DRG-related policies with earlier coverage have shown marked improvements in rehabilitation expenditures (40, 41). This “consumables substitution effect” has also been observed internationally; for instance, many hospitals have increased the use of reimbursable implants and consumables to offset reductions in conventional medical service costs (42). This indicates that, without explicit regulation on consumable usage, DRG-based payments may facilitate cost-shifting behaviors. Therefore, in the process of DRG implementation in China, it is necessary to further refine the classification and payment standards for high-value consumables to prevent the emergence of new sources of irrational cost increases. Diagnostic costs’ median dropped from 302.26 yuan pre-reform to 273.72 yuan post-reform, with reduced variance, indicating a trend toward streamlined diagnostic procedures under DRG. Although total costs slightly decreased, volatility did not significantly narrow, implying that while DRG can partly control expenses, it struggles to uniformly manage costs for complex cases. From the perspective of cost composition, DRG reform led to a marked reduction in medication costs and compression of diagnostic costs, whereas treatment and rehabilitation costs increased, possibly reflecting a policy shift from “drug-dominated” toward “technology-dominated” expenditure structures (43, 44). Total costs saw a modest decline but individual variability remained substantial, suggesting that further refinement of DRG payments, such as disease complexity grading, remains necessary to improve cost control. From an international perspective, reductions in pharmaceutical expenditures are a common outcome across most DRG systems. DRGs effectively curb the rapid growth of drug spending; however, they may also lead to an increased reliance on diagnostic and therapeutic services (45, 46). The trend observed in this study—decreased proportion of drug costs accompanied by increased proportion of technical service costs—indicates that China’s policy orientation is gradually shifting toward the value of technical services. Nonetheless, avoiding excessive technologization and consumable usage still requires careful balancing by policymakers through multiple mechanisms, including medical insurance supervision, payment standards, and clinical pathway regulations.

The analysis of structural variation through the three indicators—VSV, DsV, and CRSV—reveals the profound impact of the DRG payment reform on the inpatient cost structure of fracture patients, highlighting the complexity and specificity of medical behavior adjustments. The results not only validate the effectiveness of policy intervention but also expose structural issues worthy of attention during the reform process. First, the general decline in treatment costs (S72 group DsV = −77.62%) contrasts sharply with the significant rise in consumable costs (S72 group VSV = 3,474.02), illustrating a distinct “treatment-consumable substitution” phenomenon. This likely reflects adaptive strategies employed by medical institutions under the DRG fixed payment system. Particularly noteworthy is the extreme pattern observed in the S32 group, where diagnostic costs increased substantially (VSV = 623.70) while treatment costs plummeted (VSV = −17,123.05), potentially indicating a “substituting tests for treatment” behavior by some hospitals. This finding aligns with (20), who reported behavioral changes in diagnosis and treatment following payment reform. Second, the drastic fluctuations in consumable costs across multiple fracture types—such as the S32 group’s extraordinarily high DsV of 2,170.42%—are cause for concern. This surge may arise from two mechanisms: on one hand, there is rigid demand for high-value consumables (e.g., internal fixation devices) in orthopedic surgery (47, 48); on the other hand, it cannot be ruled out that medical institutions increase consumable usage to offset revenue losses elsewhere. While consumable regulation remains important in internal medicine where consumable proportions are low (49), greater vigilance is warranted in surgical fields with higher consumable shares to prevent regulatory evasion. The systemic decline in rehabilitation costs (S92 group VSV = −991.80) reveals potential shortcomings in the current payment policy. Contribution rate analysis shows that treatment costs dominate in many groups (CRSV > 69%), indicating that DRG exerts the strongest regulatory effect on core diagnosis and treatment activities. However, the unique “treatment-rehabilitation” dual dominance mode in the S22 group (combined CRSV > 80%) suggests that the impact of payment reform may be more multifaceted for certain fracture types.

This study, through interrupted time series analysis, confirms that the DRG payment reform has exerted a structural impact on medical costs for fracture patients. Following policy implementation, except for a rebound in some diagnostic costs, total costs, treatment costs, medication costs, and rehabilitation costs all showed a downward trend, a finding consistent with relevant domestic and international research (32). Notably, the cost containment effect was particularly pronounced in case types that previously exhibited a significant upward cost trend (e.g., S32 fractures), indicating that DRG played a key role in curbing excessively rapid growth in medical expenditures. This overall cost control effect stems from the inherent incentive for cost management created by the DRG payment standards for medical institutions. It prompts hospitals to shift their management focus forward to the starting point of the clinical pathway—the diagnostic stage. By strengthening diagnostic investment and improving classification accuracy, they establish a scientific basis for subsequent treatment plan selection, thereby reducing unnecessary medical resource consumption at the source and achieving systematic optimization of treatment costs (50). Regarding consumables costs, although they showed an overall downward trend after the DRG policy implementation, consumables costs for S42 fracture patients exhibited anomalous growth. This phenomenon may be related to the clinical characteristics of S42 fractures (mainly including clavicle, scapula, and proximal humerus fractures). The treatment options for this type of fracture are diverse, ranging from conservative treatment and closed reduction minimally invasive surgery to complex open reduction and internal fixation, and even shoulder arthroplasty. The types and costs of consumables required for different schemes vary significantly (51). After the implementation of the DRG policy, the strengthening of the diagnostic phase may prompt medical institutions to define the classification earlier and opt for more definitive surgical solutions, consequently leading to increased use of high-value consumables in S42 fractures, forming a phenomenon of “diagnosis-driven growth in consumables costs.” In terms of medication costs, Western medicine costs for all fracture types consistently decreased after the DRG policy, reflecting the significant effectiveness of the policy in standardizing medication practices. This change is related both to medical institutions actively optimizing medication regimens and promoting rational drug use, and may also benefit from the synergistic effects of the national volume-based procurement policy (52). Worthy of further exploration is that the changing trends of some TCM costs were not entirely consistent with those of Western medicine, suggesting that the use of proprietary Chinese medicines may be influenced by multiple factors such as adherence to clinical pathways and physicians’ prescribing habits. The standardized management mechanisms for TCM still require in-depth study (32). The widespread decline in treatment and rehabilitation costs demonstrates the regulatory effect of DRG on core medical services. However, phenomena such as the sharp drop in rehabilitation costs for S32 fractures (*β*₃ = −483.58) are particularly noteworthy. Although this reflects the effectiveness of cost control to a certain extent, it also hints at the potential risk that excessive compression of necessary rehabilitation services may impair patients’ functional recovery and long-term prognosis (9, 53). Therefore, while continuing to advance cost control, determining how to scientifically balance economic efficiency with medical quality will be an important direction for the future optimization of DRG policy.

The gray relational analysis revealed heterogeneity in the correlation between cost categories before and after the implementation of the DRG payment model across different fracture types. Firstly, S22 and S42 fractures showed a marked increase in the correlation coefficients of diagnostic and pharmaceutical expenses, suggesting that under DRG constraints, the relative weight of diagnostic procedures and medication management increased, consistent with standardized clinical pathways and rational drug utilization. This feature aligns with the AO principles of orthopedic internal fixation (54, 55). The trend was particularly pronounced in S42, where nearly all cost categories demonstrated steady growth, indicating that DRG implementation reinforced a balanced distribution of expenses in this fracture type. In contrast, S52 and S62 fractures exhibited an overall downward trend. In S52, the correlations for treatment, consumables, and rehabilitation decreased most notably, suggesting potential cost containment through reduced therapeutic interventions or optimized rehabilitation processes. Similarly, multiple expense categories in S62 declined significantly, implying that complex fractures may be more sensitive to cost pressures under the DRG system. By comparison, S72 fractures maintained a relatively stable structure with minimal fluctuations, suggesting little change before and after DRG or that clinical pathways were already highly standardized. S82 fractures demonstrated mild increases across categories, indicating modest optimization in expense allocation without substantial shifts. S92 fractures, however, showed consistent strengthening across all categories, suggesting that DRG exerted the most balanced and significant regulatory effect on this group. In summary, the impact of the DRG payment model on the cost structure of fracture patients was not uniform but category-specific. Diagnostic and pharmaceutical expenses increased markedly in certain fractures, whereas treatment, rehabilitation, and consumables declined in others. These differences may reflect variations in fracture complexity, the degree of clinical pathway standardization, and healthcare resource allocation.

This study is the first to integrate structural variation analysis with ITS to systematically evaluate the multi-level impacts of DRG reform on inpatient costs for fracture patients. Gray relational analysis quantified the relative contribution of different cost categories, addressing limitations of conventional statistical methods. Findings indicate that observed cost structure changes reflect not only financial effects but also the adaptive response of DRG payment in China’s evolving healthcare context. Heterogeneous responses across fracture types highlight the interplay between policy implementation and clinical practice, offering empirical evidence to guide policy refinement. While DRG reform enhanced overall cost control, behavioral responses such as upcoding and selective admission may affect outcome validity, and older population with multiple comorbidities experienced greater shifts in cost structure, suggesting potential under-treatment risks.

Several limitations warrant consideration. Data were derived from a single region, potentially limiting generalizability, and functional outcome measures, frailty indices, and comorbidity severity scores were unavailable, constraining evaluation of cost-quality relationships. The study period overlapped with the COVID-19 pandemic, which may have transiently influenced healthcare utilization and resource allocation. Additionally, only cost indicators were analyzed, without integrating functional recovery, readmission, or mortality, precluding full assessment of cost-effectiveness. Some fracture subgroups had limited sample sizes, which could mask subtle variations. Future multi-center, longitudinal studies incorporating clinical outcomes and quality-of-life measures are needed to more comprehensively assess the effectiveness and equity implications of DRG reform.

However, our analysis was limited to cost indicators and did not incorporate clinical outcomes such as functional recovery, readmission, mortality, or patient-reported experience measures, which precludes a full assessment of cost-effectiveness. Future multi-center, longitudinal studies integrating these broader indicators are needed to more comprehensively evaluate the effectiveness and equity implications of DRG reform. In summary, while DRG reform shows promise in controlling costs, achieving a balance between economic efficiency, clinical effectiveness, and patient-centered care will be essential for its long-term success.

## 5 Conclusion

The DRG-based payment reform has effectively controlled pharmaceutical expenditures and increased diagnostic costs, consequently triggering structural shifts in the treatment of certain fracture types, such as increased use of medical consumables and reduced treatment intensity. The reform outcomes exhibit marked fracture-type specificity, highlighting the necessity for implementing differentiated and refined management strategies. This study provides empirical evidence to support the optimization of DRG payment standards and offers valuable insights for advancing the reform of the healthcare payment system.

## Data Availability

The data supporting the conclusions of this article will be made available by the authors, upon reasonable request to the corresponding author.
